# Combining field performance with controlled environment plant imaging to identify the genetic control of growth and transpiration underlying yield response to water-deficit stress in wheat

**DOI:** 10.1093/jxb/erv320

**Published:** 2015-07-15

**Authors:** Boris Parent, Fahimeh Shahinnia, Lance Maphosa, Bettina Berger, Huwaida Rabie, Ken Chalmers, Alex Kovalchuk, Peter Langridge, Delphine Fleury

**Affiliations:** ^1^Australian Centre for Plant Functional Genomics (ACPFG), University of Adelaide, PMB 1, Glen Osmond, SA 5064, Australia; ^2^The Plant Accelerator, Australian Plant Phenomics Facility, University of Adelaide, PMB 1, Glen Osmond, SA 5064, Australia; ^3^University of South Australia, GPO Box 2471, Adelaide, SA 5001, Australia; ^4^School of Agriculture, Food and Wine, University of Adelaide, PMB 1, Glen Osmond, SA 5064, Australia

**Keywords:** Drought, leaf expansion, Lemnatec, QTL, *Triticum aestivum*, water-use efficiency.

## Abstract

Crop yield in low-rainfall environments is a complex trait under multigenic control that shows significant genotype×environment (G×E) interaction. One way to understand and track this trait is to link physiological studies to genetics by using imaging platforms to phenotype large segregating populations. A wheat population developed from parental lines contrasting in their mechanisms of yield maintenance under water deficit was studied in both an imaging platform and in the field. We combined phenotyping methods in a common analysis pipeline to estimate biomass and leaf area from images and then inferred growth and relative growth rate, transpiration, and water-use efficiency, and applied these to genetic analysis. From the 20 quantitative trait loci (QTLs) found for several traits in the platform, some showed strong effects, accounting for between 26 and 43% of the variation on chromosomes 1A and 1B, indicating that the G×E interaction could be reduced in a controlled environment and by using dynamic variables. Co-location of QTLs identified in the platform and in the field showed a possible common genetic basis at some loci. Co-located QTLs were found for average growth rate, leaf expansion rate, transpiration rate, and water-use efficiency from the platform with yield, spike number, grain weight, grain number, and harvest index in the field. These results demonstrated that imaging platforms are a suitable alternative to field-based screening and may be used to phenotype recombinant lines for positional cloning.

## Highlight

We describe new quantitative trait loci for growth and transpiration in wheat under two water regimes using an imaging platform, and co-location with loci for yield components in the field.

## Introduction

Drought is a major cause of decreased crop production worldwide. In Australian dryland agriculture, grain crop yields are approximately 50% of their potential and are highly unpredictable. The 2006 drought reduced the total Australian wheat (*Triticum aestivum* L.) yield by 46% (FAO, 2013). During the 1990s, the rate of productivity increase in Australian broadacre cropping improved by 3.4% annually but has since slowed to about 1.4%. Yield is the end product of a grain crop, integrating the genetic ability of the plant to grow, assimilate carbon, and transfer it to the grain, and the effects of environmental conditions on these different plant processes. Yield is therefore a complex trait under multigenic control and is highly influenced by genotype×environment (G×E) interactions (Tardieu, 2010).

Although many quantitative trait loci (QTLs) have been identified in wheat for yield and yield components in low-yielding, rain-fed environments (reviewed by Fleury and Langridge, 2014), the underlying genes have yet to be cloned. Many QTLs called ‘unstable’ across environments are observed under specific environmental conditions only (G×E interactions). For example, the allele carried by the RAC875 parental line at the QTL for yield on chromosome 3B in wheat is only positive in hot and dry environments where the soil is deep, such as in northern Australia and Mexico, but not in southern Australia (Bonneau *et al.*, 2013). In addition, gene cloning in wheat is still a difficult task due to the size (17 Gb) and complexity of the genome: the bread wheat genome is hexaploid with three homeologous A, B, and D genomes, and contains about 80% repeat sequences (Smith and Flavell, 1975; Choulet *et al.*, 2010). The availability of genomic resources has substantially increased in recent years, significantly enhancing progress in genetic mapping and gene identification (Periyannan *et al.*, 2013; Saintenac *et al.*, 2013). As the availability of these resources becomes commonplace, phenotypic screening has become a bottleneck in the understanding and tracking of complex phenotypes such as yield in dry environments.

While plant physiologists have made progress in understanding the mechanisms of drought tolerance, this has not translated into tools that can be effectively utilized by breeding programmes. Tardieu and Tuberosa (2010) suggested that dissecting complex traits into simple components independent of many confounding environmental effects could be undertaken in highly controlled phenotyping platforms. The development of high-throughput phenotyping platforms over the last 10 years has improved evaluation of large genetic populations (Berger *et al.*, 2010). It is now possible to apply these technical advances to the fine-mapping and possible cloning of genetic loci underlying QTLs.

The imaging platform of The Plant Accelerator at the Australian Plant Phenomics Facility and University of Adelaide, Australia, allowed us to image, weigh, and water thousands of plants every 2 d (Honsdorf *et al.*, 2014). We developed routines to convert pixels from red/green/blue images into biomass and leaf area, to infer growth and relative growth rate, transpiration, and water-use efficiency (WUE) and applied these techniques to the analyses of a recombinant inbred line (RIL) population under different watering regimes.

The selected genetic population was a set of RILs derived from a cross between Drysdale and Gladius, two modern bread wheat varieties adapted to the southern region of the Australian wheat belt, characterized by a Mediterranean-type climate. As a consequence of the limited water storage in these soils, crops rely on season rainfall, which becomes sporadic in spring, leading to cyclic drought from the heading stage until the end of grain filling. Both Gladius and Drysdale perform well in a low-to-medium-rainfall environment but show different mechanisms of response to drought (Fleury *et al.*, 2010). Gladius is an erect and waxy leaf variety selected for yield under severe drought in South Australia. Drysdale is a transpirationally efficient variety, which was selected for high carbon isotope discrimination, a surrogate for transpiration efficiency (Rebetzke *et al.*, 2002; Condon *et al.*, 2006). Segregation of these traits in Drysdale×Gladius progeny makes this population an interesting resource for dissecting the genetics of physiological mechanisms of adaptation to drought.

In this study, we used a genetic population with parental lines contrasting in their mechanisms of yield maintenance under water deficit. We analysed plant growth, transpiration, and WUE from imaging and watering data from a phenotyping platform for plants grown under two different watering regimes. The results were compared with QTLs identified for yield components in a field environment controlled for temperature and the watering regime. The comparison led to the discovery of co-located QTLs for plant growth, transpiration, and yield components, indicating that imaging platforms can be used to phenotype recombinant lines.

## Materials and methods

### Plant material

Genetic analysis was undertaken in a RIL population of 5000 lines from a cross between Drysdale and Gladius, two spring wheat varieties with the following respective pedigrees: Hartog×3 Quarrion for Drysdale, and RAC875/Krichauff//Excalibur/Kukri/3/RAC875/Krichauff/4/RAC875//Excalibur/Kukri for Gladius. Four sets of lines were used for different purposes. Set 1 consisting of 250 randomly chosen RILs was used for the simple sequence repeat (SSR) and diversity arrays technology (DArT) genetic map construction and phenotyping under semi-controlled field conditions in polyurethane tunnels (polytunnels) in 2010. A subset of 150 lines (set 2) flowering in a 6 d window was chosen from set 1 in order to apply the drought stress at a similar stage of development. This set was complemented with 100 randomly chosen RILs to construct the single nucleotide polymorphism (SNP) genetic map (set 3). The 115 RILs (set 4) common between sets 1 and 3 were genotyped to construct an integrated SSR, DArT, and SNP map.

### Field trials

Two experiments were carried out under semi-controlled field conditions in the polytunnel facility of the University of Adelaide (Urrbrae, South Australia, Australia, 35° S 139° E). This facility includes bird nets and polytunnels, equipped with automatic watering systems (drippers) and weather stations (MEA, Adelaide, Australia) recording air temperature and humidity at 2 m height and at the plant canopy level, as well as soil temperature and wind. Gypsum blocks (MEA) were used for measuring soil water tension at three different soil depth (15, 30, and 40cm from the soil surface, eight sensors per watering regime). All climatic data were averaged and stored every 10min in a data logger (MEA).

### Plant growth conditions

In 2010, 250 RILs of set 1 were grown under well-watered and water-deficit treatments. This trial was sown on 9 June, later than the normal commercial sowing time (April/May) for wheat in South Australia, in order to expose plants to drought stress during flowering and grain filling. In both treatments, micro-plots (10×120cm, 16 plants, density of 133 plants m^–2^) were randomly distributed and partially replicated (two replicates for 60 RILs and three replicates for the parental lines). On both sides of each lane of micro-plots, a micro-plot was sown with genotype Gladius (not analysed) to prevent any border effect. Both treatments were sown under bird nets and maintained as well watered (soil water tension > –0.1MPa) during vegetative growth using a dripper system. In the drought treatment, watering was stopped at stem elongation, and a polytunnel was installed as a rain shelter. When the soil dried below –0.6MPa, a light watering of 15mm was applied three times before harvesting the plants. In the well-watered treatment, watering was maintained until 15 d after flowering. Plants were fertilized (Aquasol, Hortico, Clayton, VIC, Australia) twice in both treatments: at stem elongation stage and at flowering stage. A fungicide (Bayfidan; Bayer Australia, Pymble, NSW, Australia) was applied at booting and flowering stages.

Plants experienced similar shoot micro-environments in both water regimes, with air temperature from 3.8 to 31 °C (average 12.4 °C) and a maximum vapour pressure deficit (VPD) of 3.5 kPa. Soil water tension in the well-watered treatment stayed above –0.1MPa until 15 d_20°C_ (equivalent day at 20 °C, explained in ‘Thermal compensation of time and rates’) after flowering when plants experienced a moderate water deficit (> –0.4MPa) for 10 d. In the water-deficit treatment, the soil started to dry when irrigation was stopped and reached –0.6MPa around 5 d_20°C_ after flowering. The soil water tension was then kept around –0.6MPa with light watering.

In 2011, 148 lines of the Drysdale/Gladius RIL set 2 were grown in the same platform under two water regimes: well watered and water deficit. This experiment was sown on 14 July, with micro-plots of 40×60cm (32 plants, 133 plants m^–2^). Both treatments were well watered until stem elongation. Then, watering was stopped in the drought treatment and lightly watered as in 2010. Urea (Manutec, Cavan, SA, Australia) was mixed with the top soil before sowing (N rate 225kg ha^–1^); the fertilizer and fungicide regime during plant growth was the same as in 2010.

In both water regimes, air conditions were warmer and dryer than in 2010, due to the later sowing, with air temperature from 4.8 to 35.2 °C (average 15.2 °C) and a maximum VPD of 4.9 kPa. Soil water potential stayed above –0.1MPa until 10 d_20°C_ before flowering in both treatments and then started to dry at different speeds depending on treatment. Soil water potential reached –0.6MPa around flowering in the water-deficit treatment and around 20 d_20°_C after flowering in the well-watered treatment.

### Plant measurements

*Heading time* (d; six first spikes appeared in the plot), and *Flowering time* (d; six spikes flowering on one-third of the spike length) were scored every day from the first spike appearance. All tillers were manually harvested, and the number of tillers per plant (*Tiller number*) and spikes per plant (*Spike number*) were counted. After drying (10 % moisture content), *Stem weight* (g), *Grain weight* (g), and total plant weight (*Biomass*, g) were measured and calculated per plant. A sample of 100ml of seed was weighed, and the seeds were counted with a seed counter (Pfueffer GmBH, Germany) to estimate *Single grain weight* (g) and total *Seed number* per plant. Grain weight per plot was converted to *Yield* (t ha^–1^) for an easier comparison with other trials. *Harvest index* was calculated as the ratio of *Grain weight*/*Biomass*.

### Imaging platform experiment

#### Plant growth conditions

An experiment with 150 RILs of set 2 was performed in The Plant Accelerator (Honsdorf *et al.*, 2014) greenhouse facilities in Urrbrae, South Australia, Australia (34°58′16.18″ S; 138°38′23.88″ E). Two watering regimes were applied (well watered and stable drought), and each line was replicated twice. Well-watered and drought-stressed plants of the same line and replicate number were placed next to each other. Single plants were grown in 2.5 l plastic pots filled with 3kg of potting mix (50% coco/peat mix, 50% clay/loam). Three seeds per pot were sown and thinned to one plant per pot at the three-leaf stage. Two pots per treatment contained artificial plants and were placed in the middle of the greenhouse, under the same watering regimes as pots with plants in order to estimate the evaporation from the soil surface.

During the first 3 weeks, plants were manually well- watered (> –0.02MPa) in both treatments. Plants were then transferred to the imaging platform of The Plant Accelerator, where each pot was placed onto a cart on a conveyor belt until flowering. Plants were imaged using a LemnaTec 3D Scanalyzer (LemnaTec, GmbH, Aachen, Germany). Three red/green/blue images (2056×2454 pixels) were taken with top and two side views with a 90° horizontal rotation (Honsdorf *et al.*, 2014). Background–foreground separation was then applied to separate the plant tissue area from the background, and pixel numbers per image were counted after noise removal.

Environmental conditions fluctuated naturally in the greenhouse as plants experienced natural lighting and temperature ranking from 17 °C (night) to 25 °C (day). Every second day, pots were automatically weighed and watered to –0.02 and –0.05MPa (well-watered and drought treatments, respectively). Soil water content was measured by automatically weighing the pots. Differences in weight were attributed to changes in soil water content, after correction for the increase in plant *Biomass* mean (see below). A water-release curve of the soil was obtained on additional pots. Five pots containing three plants were dried from soil water retention capacity to –1.6MPa. After long nights (>12h) in a growth chamber with air saturated with water, pre-dawn leaf water potential was measured on non-expanding leaves using a Scholander-type pressure chamber (Soil Moisture Equipment Corp., Santa Barbara, USA). A Van Genucheten curve (Van Genuchten, 1980) was fitted to these data (soil water potential vs soil water content), thereby allowing calculation of the mean soil water potential in each pot at each weighing time (Supplementary Fig. S1, available at *JXB* online). Water loss per pot between two watering events was considered as plant *Transpiration* after correcting for soil evaporation measured using pots with artificial plants.

#### Calibration by plant destructive measurements

A separate experiment with similar conditions of culture was carried out on the two parental lines in order to convert pixel values obtained by image analysis into biological variables. From 2 weeks after sowing to flowering, three plants per genotype and per treatment were harvested twice a week. Plants were directly weighed (*Plant weight*, g). *Leaf area* (mm^2^) was measured with a planimeter (PATON electronic belt driven planimeter, CSIRO, Canberra, Australia). *Biomass* (g) was measured after 1 week at 65 °C.

### Data analysis

Analysis of variance (ANOVA) and correlation analysis was performed for data obtained from different experiments using PROC GLM and PROC CORR, respectively, in SAS Software v.8.1 (SAS Institute, 2000). Broad-sense heritability (*h* ^2^) was calculated from variance components according to Kearsey and Pooni (1996). Data obtained from the imaging platform was analysed using R Software (R Development Core Team, 2013). Non-linear models were fitted with the *nls* function of the R package.

### Thermal compensation of time and rates

Time and rates were expressed as thermal time as described by Parent *et al.* (2010) and Parent and Tardieu (2012). Briefly, the temperature responses of development processes were described by the equation of Johnson *et al.* (1942), modified by Parent and Tardieu (2012), and applied in different studies of developmental processes (Parent *et al.*, 2009, 2010; Louarn *et al.*, 2010):

$$F(T)=\frac{ATe^{\left(\frac{-\Delta H_{A}^{{}^{‡}}}{RT}\right)}}{1+{\left[e^{\left(\frac{-\Delta H_{A}^{{}^{{}^{‡}}}}{RT}\right)}\right]}^{\alpha \left[1-\frac{T}{T_{0}}\right]}}$$ (1)

where *F*(*T*) is the considered rate, *T* is the temperature (K), ∆H_A_^‡^ (J mol^–1^) is the enthalpy of activation of the process and determines the curvature at low temperature, *α* (dimensionless) determines how sharp the rate decrease is at high temperature and is fixed at 3.5 for developmental processes (Parent and Tardieu, 2012), T_0_ (K) determines the temperature at which the rate is maximum, and A is the trait scaling coefficient.

For any measured rate *J*(*T*) at temperature *T*, a temperature compensated rate was calculated as the equivalent rate at 20 °C.

$$J_{20{}^{\circ}\text{C}}=J(T)\frac{F\left(20{}^{\circ}\text{C}\right)}{F(T)}$$ (2)

with *F*(*T*) being the response of development to temperature. As developmental time (or thermal time *t*_20°C_) is the reciprocal of development rate, it results in:

$$t_{20{}^{\circ}\text{C}}=t(T)\frac{F\left(T\right)}{F(20{}^{\circ}\text{C})}$$ (3)

with *t*_20°C_ being expressed either as equivalent hour at 20 °C (h_20°C_) or equivalent day at 20 °C (d_20°C_), depending on the unit of *t.*

### Analysis pipeline for platform data

An analysis pipeline was developed to convert pixels from platform images into variables of biological interest such as *Biomass*, *Plant weight*, *Leaf area*, *Average growth rate* or *WUE.*

#### Converting pixels into projected areas by using simple trigonometric equations

A length measured in pixels (*side.distance*) from a side-view image can be converted to a distance in mm with a single coefficient (side.coeff), the size of a pixel:

$$\begin{array}{l} side.distance\left(\text{mm}\right)=side.distance\\ \;\left(\text{pixel}\right)\times \text{side}.\text{coeff }\left({\text{mm pixel}}^{–\text{1}}\right) \end{array}$$ (4)

with side.coeff=0.5156mm pixel^–1^ for the camera settings used.

For a top-view image, this coefficient (*top.coeff*, mm pixel^–1^) depends on plant height. First, the size of a pixel is calculated depending on the distance between the object and the camera:

top.coeff=top.slope×cm.pix+top.offset (5)

with top.slope (0.0001097mm pixel^–2^) and top.offset (0.2899mm pixel^–1^) as the linear parameters of the relationship and *cm.pix* (pixel) as the *y*-coordinate of the centre of mass of the plant obtained from the side view images.

The projected areas can then be calculated as:

$$side.area\left(mm^{2}\right)=side.area\left(pixel\right)\times side.coeff^{2}$$ (6)

$$top.area\left(mm^{2}\right)=top.area\left(pixel\right)\times top.coeff^{2}$$ (7)

*Linear models converting projected areas to* Biomass, Plant weight, *and* Leaf area For each biological variable (*Biomass*, *Plant weight*, or *Leaf area*), the complete linear model with projected areas at the second order was tested, as well as all derived simpler models on data from the second experiment with destructive measurements. The complete model is:

$$\begin{array}{l} Y=side.area+top.area+side.area^{2}\\ \;+top.area^{2}+side.area:top.area \end{array}$$ (8)

These models were compared using the Bayesian information criterion (BIC) and the model with the lowest value was selected.

**Fig. 1.**
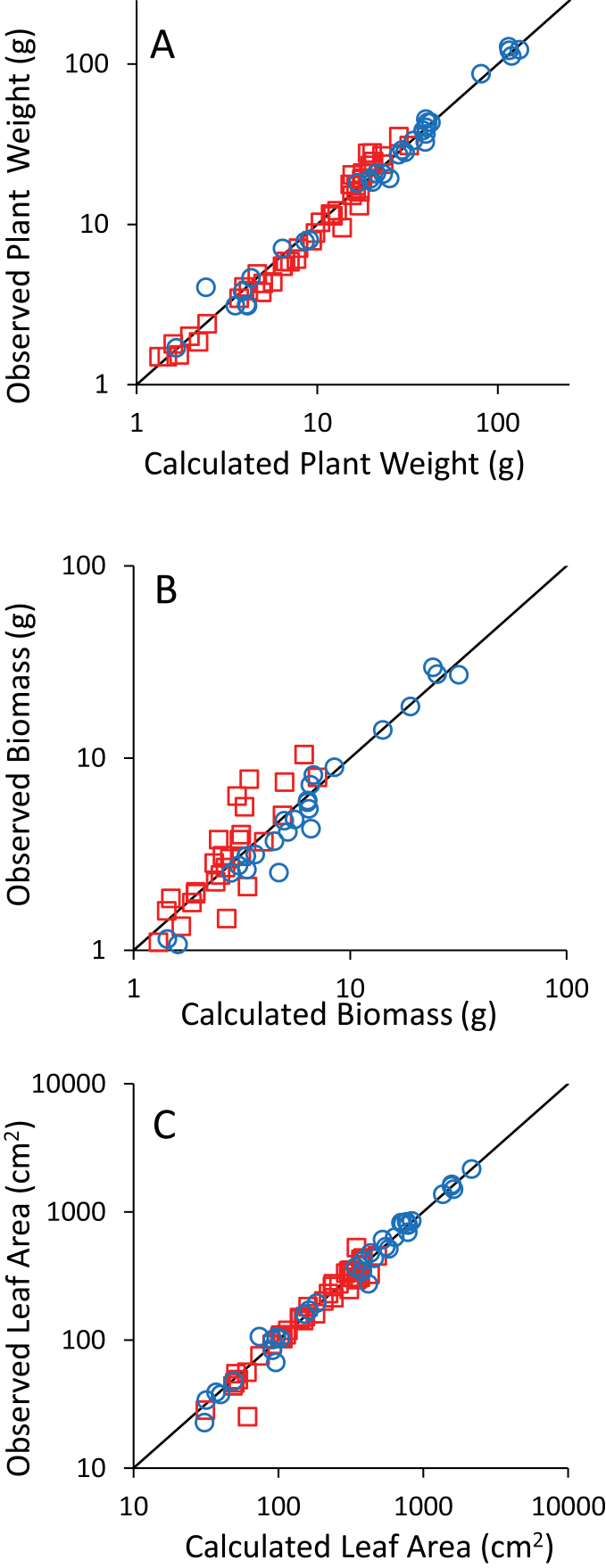
Plots of observed/calculated variables for *Plant weight* (A), *Biomass* (B), and *Leaf area* (C). Circles are data for well-watered plants and squares are for drought-stressed plants. The line is the 1:1 line. The scale is logarithmic for better data visualization but models were selected on raw data. (This figure is available in colour at *JXB* online.)

This procedure produced similar results for *Biomass* and *Leaf area* but different results for *Plant weight* (Table 1). The models were used to infer biological variables with the same parameters for well-watered plants and drought-stressed plants, but the error was higher for *Biomass* than for *Plant weight* and *Leaf area* (Fig. 1). It was decided to keep the same model for plants under well-watered and water-deficit conditions to better compare the treatments and to allow the calculation of variable responses to soil water potential.

**Table 1.** Models selected for inferring Leaf Area, Plant Weight and Biomass from projected areas Side and top are the average projected shoot area (mm^2^) on the side view and the projected shoot area on the top view (mm^2^), respectively. Models have been selected with BIC. ***P<0.001, **P <0.01, and (.), *P*<0.1 in an ANOVA test. Where no sign is given, this predictor was not selected by the BIC test.

Intercept	Leaf area	Plant weight	Biomass
Side	***	***	***
Top	(.)		***
Side^2^	**	***	**
Top^2^	***		***
Side:top	***	(.)	***

#### Fitting growth curves to experimental data

**Fig. 2.**
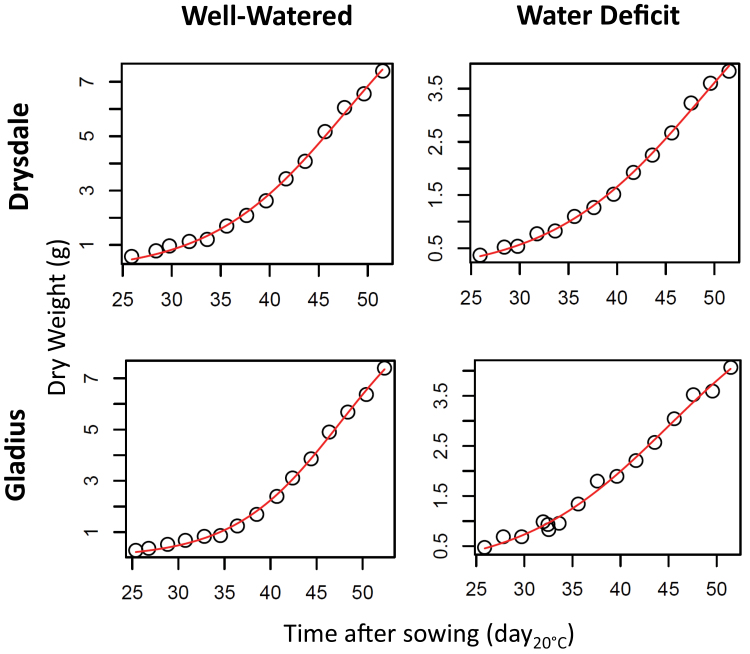
Growth curves for calculated *Biomass* over thermal time in parental lines. Growth curves were calculated on single plants and these plots are examples of single plants. Circles indicate the calculated data. The solid line indicates the logistic (three-parameter) models. (This figure is available in colour at *JXB* online.)

Different models for fitting *Biomass*, *Plant weight*, or *Leaf area* over time have been tested with the R function for non-linear regression (*nls*(), with our own self-start functions) on the two parental lines Gladius and Drysdale: exponential, linear, logistic with three (Chen *et al.*, 2014) or four parameters, thye equation of Richards with four or five parameters, Gompertz with four parameters (Chen *et al.*, 2014), and Weibull with three or four parameters. Some models were not adapted to all datasets. Only the linear, exponential, and logistic three parameters converged for all plants, but the logistic equation fitted best (BIC tests; results not shown) and was therefore applied to all data (Fig. 2).

$$Variable=\;\frac{Variable_{final}}{1+e^{-K(t-t_{0)}}}$$ (9)

In this equation, *t*_0_ is the inflexion point and is considered as the transition from the vegetative to the reproductive stage. *K* is the *Maximum relative growth rate* (K_RGR_).

A logistic curve was fitted for each plant for *Biomass*, *Plant weight*, and *Leaf area* with time expressed as d_20°C_. This model for *Biomass*, *Plant weight*, and *Leaf area* fitted well with experimental data, even for *Biomass* (Fig. 2).

#### Water-use calculations

Soil water potential was calculated at each date using a water-release curve (Supplementary Fig. S1), measuring pot weight and calculating *Plant weight* using the logistic equation applied to plant images.

*Average transpiration rate* (TR, g d_20°C_^–1^) was calculated from water loss between days 35 and 50 after sowing (vegetative stage in all genotypes), taking into account the soil evaporation and plant weight. *Average transpiration rate per unit of leaf area* (TR_area_, g mm^–2^ d_20°C_^–1^) was calculated from the leaf area inferred by the growth model:

$$TR_{area}=\left(\frac{water\;loss-soil\;evaporation}{leaf\;area}\right)$$ (10)

#### Derived variables such as Growth, RGR, and WUE

For the three variables (*Biomass*, *Plant weight*, or *Leaf area*), their growth rate and *Average relative growth rate* (RGR) were derived from their growth curve:

$$Growth\;rate=\;\frac{dVariable}{dt}$$ (11)

$$RGR=\;\frac{growth\;rate}{variable}$$ (12)

In this analysis, *Growth* (g d_20°C_^–1^) is the increase of biomass with time, and *Average leaf expansion rate* (LER, mm^2^ d_20°C_^–1^) is the increase of leaf area. *RGR* (d_20°C_^–1^) is the relative increase of biomass and *Average relative leaf expansion rate* (RER, d_20°C_^–1^) is the relative increase of *Leaf area*. *Average growth rate* (Growth._AVE_, g d_20°C_^–1^) and *Average leaf expansion rate* (LER_AVE_, mm^2^ d_20°C_^–1^) were calculated from day 35 to day 50.

WUE (g g^–1^) was calculated as the *Average growth rate* divided by transpiration from day 35 to day 50.

$$WUE=\frac{growth\;rate}{water\;loss-soil\;evaporation}$$

For all of these variables, their relative response to soil water potential (Ψ) was calculated as:

Relative response to soil water potential=VariableD−VariablewwΨD− ΨWW (13)

with *Variable*_WW_ and *Variable*_D_, respectively, being the value of the considered biological variable in well-watered and drought conditions and *Ψ*_WW_ and *Ψ*_D_, respectively, being the value of Ψ in well-watered and drought conditions.

### Genetic map construction

DNA was extracted from 2.0g of bulked leaf tissue (three to six plants per line) of 8-week-old plants using a mini prep ball bearing extraction method with minor modifications (Pallotta *et al.*, 2000). The RIL sets 1 and 3 were genotyped with markers for genes that control phenology in wheat: vernalization genes *Vrn-A1* and *Vrn-D1*, and photoperiod genes *Ppd-B1* and *Ppd-D1*, as described by Maphosa *et al.* (2014). For each line to be genotyped with SNP markers (set 3), approximately 100ng μl^–1^ of DNA (30 μl) was sent to the Department of Primary Industries, Victoria, to be assayed on an 9k SNP iSelect BeadChip array as described by Akhunov *et al.* (2009).

Three linkage maps were used. The first was based on DArT, SSR, and gene-based markers on RIL set 1 (Maphosa *et al.*, 2014). The second map was constructed using SNP and gene-based markers on RIL set 3, which consisted of a mixture of random and a selection of mid-maturing lines. The third map was based on DArT, SSR, and SNP using RIL set 4. The method of genetic map construction was described by Maphosa *et al.* (2014).

### QTL mapping

QTL analysis was performed for the mean spatial adjusted value of traits using PROC GLM, SAS software (v.9). Initially, single marker analysis was used for each trait to identify markers associated with variation for traits. Further evaluation was carried out by composite interval mapping with a 15 cM window and a maximum of 15 marker co-factors per model using Windows QTL Cartographer version 2.0 (Wang *et al.*, 2004). As the 150 lines of set 2 were still segregating for the *Vrn-A1*, *Vrn-D1*, *Ppd-B1*, and *Ppd-D1* genes (Beales *et al.*, 2007; Zhang *et al.*, 2008), the model was adjusted to remove their effect on growth and yield. Tests were performed at 1 cM intervals, and effect of these genes was selected as the co-factor by forward–backward stepwise regression (Model 6). Genome-wide, trait-specific threshold values (*α*= 0.05) of the likelihood ratio test statistic for declaring the presence of a QTL were estimated from 1000 permutation tests by random sampling of phenotypic data (Churchill and Doerge, 1994; Doerge and Churchill, 1996). The phenotypic variation explained by a QTL (*R* ^2^) conditioned by the composite interval mapping co-factors included in the model was calculated at the most likely QTL position. The additive effect of an allelic substitution at each QTL was also obtained. The log of odds (LOD) peak of each significant QTL was considered as the QTL location on the linkage map.

The QTL analysis of the imaging platform experiment on RIL set 2 was done using the SNP map. All QTL intervals were located on the SSR–DArT–SNP map and the SNP map for comparing the position of QTLs for different traits and platforms.

## Results

### Analysis of the imaging platform data

*Biomass*, *Plant weight*, *Leaf area*, *Average growth rate*, and *WUE* were calculated using the new analysis pipeline. The same pipeline was adapted to plants for all genotypes and treatments (Fig. 2). These data were then used for genetic analysis. Transgressive segregation and significant genetic variation among the lines was observed for most of these traits (Table 2). As expected, large differences were observed between the two watering regimes, so each treatment was analysed separately. The broad-sense heritability (*h* ^2^) values ranged from 0 to 54%, indicating that the proportion of genetic-to-environmental variation differed among traits.

**Table 2.** Descriptive statistics and heritability (h^2^, %) for the traits measured on the 150 Drysdale/Gladius RIL phenotyped in the imaging platform SD, standard deviation; Min, minimum, Max, maximum; ***P<0.001, **P<0.01, *P <0.05 and NS, *P* >0.05 (not significant) in an ANOVA test.

Variable		Well watered				Drought			
Name	Acronym	Mean±SD	(Min – Max)	*h* ^2^	*P*	Mean±SD	(Min – Max)	*h* ^2^	*P*
*Average growth rate*	Growth_AVE_ (g d_20°C_^–1^)	0.332±0.076	(0.108–0.533)	40.8	***	0.121±0.047	(0.032–0.27)	51.1	***
*Average relative growth rate*	RGR (d_20°C_^–1^)	0.102±0.008	(0.076–0.126)	5.3	NS	0.075±0.014	(0.038–0.117)	46.1	***
*Inflexion point in growth curves*	Tx_growth_ (d_20°C_)	56.11±2.83	(50.43–65.14)	6.6	NS	51.41±7.75	(38.84–97.06)	43.3	***
*Maximum relative growth rate*	K_RGR_ (d_20°C_^–1^)	0.143±0.014	(0.113–0.188)	3.2	NS	0.130±0.023	(0.079–0.215)	30.0	***
*Average leaf expansion rate*	LER_AVE_ (mm^2^ d_20°C_^–1^)	3062±725	(997–4842)	37.4	***	999±364	(329–2137)	41.1	***
*Average relative leaf expansion rate*	RER (d_20°C_^–1^)	0.092±0.007	(0.072–0.110)	35.7	***	0.056±0.010	(0.031–0.084)	47.6	***
*Maximum relative leaf expansion rate*	K_RER_ (d_20°C_^–1^)	0.122±0.010	(0.086–0.146)	24.4	***	0.102±0.014	(0.070–0.139)	53.8	***
*Average transpiration rate*	TR (g d^–1^)	88.9±16.0	(46.6–123.2)	14.1	*	38.2±7.1	(25.1–78.7)	22.2	**
*Average transpiration rate per unit leaf area*	TR_area_ (g mm^–2^ d_20°C_^–1^)	2.98±0.47	(1.24–4.68)	11.9	*	2.08±0.37	(1.40–3.61)	25.7	**
WUE	WUE (g g^–1^)	0.003±0.001	(0.002–0.006)	21.7	***	0.003±0.001	(0.001–0.005)	44.6	***

We compared the data from the 2011 experiments in semi-controlled field conditions (polytunnel) and from the platform experiment, both performed on the same RIL. No significant correlations were found between traits measured in the imaging platform in pots (growth, transpiration, and WUE) and traits measured in the polytunnel (yield, yield components, and phenology), with *r* values ranging from –0.3 to 0.2 (Supplementary Fig. S2, available at *JXB* online).

### QTLs for plant growth using the imaging platform

Using a genetic map of 3200 SNP markers and 783 loci, a total of 21 QTLs were identified for the platform variables in the Drysdale/Gladius population (Table 3). We identified a total of 14 QTLs for traits related to plant growth in both water treatments. Four QTLs showed strong effects ranging from 16 to 43% of the genetic variation of the trait on chromosomes 1A and 1B. Growth_AVE_ was controlled by four QTLs, which altogether explained 61% of the variation. Six QTLs for traits related to transpiration were identified.

**Table 3.** QTLs for traits measured using the imaging platform The additive effect is expressed in specific trait units. A positive value means that the trait increase is due to the Drysdale allele, while a negative value indicates the Gladius allele. Chr, chromosome. *R* ^2^ is the percentage of the genetic variation of the trait explained by the QTL.

Trait	QTL	Chr	LOD threshold^*a*^	Marker interval	QTL (cM)	LOD	Additive effect	*R* ^2^ (%)
Well-watered								
Growth_AVE_	QGRO.atw-1B	1BL	3.3	Ex_c5296_9365847	68.2	6.2	+0.030	43
				CAP7_c4778_2155754				
	QGRO.atw-2A.1	2AS	3.3	JD_c18695_17091254	52.1	4.3	+0.025	9
				Ex_rep_c66709_65042923				
	QGRO.atw-2A.2	2AL	3.3	BF475068A_Ta_2_1	63.2	3.8	–0.010	5
				Ex_rep_c69799_68761171				
	QGRO.atw-5A	5AL	3.3	Ex_c32414_41076471	8	5.3	+0.060	4
				Ex_c2505_4679749				
LER_AVE_	QLER_AVE_.atw-1B.1	1BL	3.5	Ex_c5296_9365847	68.8	4.4	+242	26
				CAP7_c4778_2155754				
	QLER_AVE_.atw-1B.2	1BL	3.4	Ex_c5296_9365847	60.6	6.4	–304	16
				CAP11_c1902_1022590				
RER	QRER.atw-1A	1AL	3.6	Ra_c2227_4304970	50.3	3.6	+0.002	30
				Ex_c15377_23637176				
TR	QTR.atw-1B	1BL	3.1	Ex_c5296_9365847	72.8	4.9	+6.2	15
				CAP7_c4778_2155754				
TR_area_	QTR_area_.atw-2D	2DL	2.9	Ex_rep_c69782_68740893	38.1	3	+0.036	3
				Ra_c3057_5773026				
**Drought**								
Growth_AVE_	QGRO.atd-5B	5BL	3.3	Ex_c35398_43558614	111.1	4.8	–0.020	9
				Ex_c11951_19164786				
RGR	QRGR.atd-4A	4AL	3.4	Ex_c5487_9686018	120.8	3.4	–0.001	10
				Ex_c14478_22481430				
Tx_growth_	QTx_growth_.atd-7D	7DS	2.8	Ex_c17914_26681837	3.5	3.2	+2.75	10
				Ex_c11813_18968198				
LER_AVE_	QLER_AVE_.atd-5B	5BL	3.3	Ex_c35398_43558614	112.4	4.4	–130	10
				Ex_c11951_19164786				
Tx_LER_	QTx_LER_.atd-5B	5BL	3.3	Ex_c35398_43558614	113.6	3.6	–2.7	6
				Ex_c11951_19164786				
TR	QTR.atd-3A	3AL	2.8	Ex_c11877_19055556	97	31	+2.1	2
				Ex_c15674_24004810				
	QTR.atd-4B	4BL	2.8	Ex_c28687_37791888	61.6	4.6	–2.6	3
				Ex_c17211_25859780				
WUE	QWUE.atd-2A	2AL	3.0	BE406351A_Ta_2_3	66.5	4	–0.0003	3
				Ex_rep_c69799_68761171				
**Relative response to soil water potential**								
K_RGR_	QK_RGR_.atr-5A	5AS	3.3	JD_c5795_6955031	32.9	3.9	–0.2	3
				Ra_c8898_14972290				
K_RER_	QK_RER_.atr-3A	3AL	3.1	Ex_c11910_19101291	67.2	4.1	–0.1	2
				Ku_c38911_47455674				
TR_area_	QTR_area_.atr-6A	6AS	1.8	CAP12_c1663_836753	6.7	2	+0.2	10
				Ex_c965_1846161				

^*a*^ The LOD threshold was empirically estimated at *α*= 0.05 from 1000 permutation tests by random sampling of phenotypic data for each trait.

**Fig. 3.**
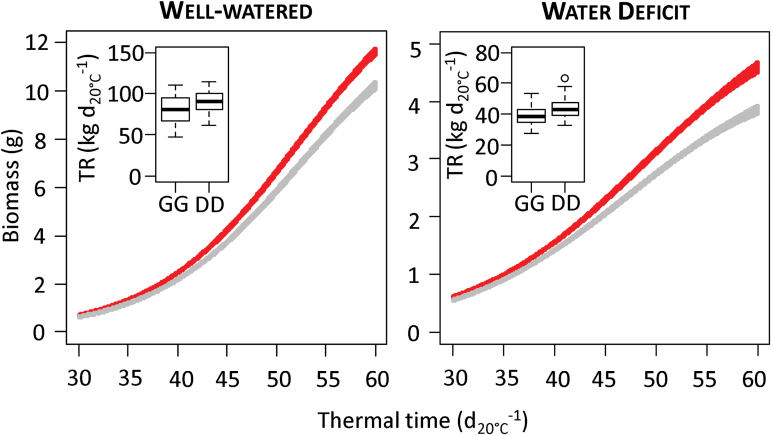
Difference between lines with the Drysdale or Gladius alleles at marker wsnp_CAP11_c1902_1022590 (position 74.3 on chromosome 1B) for their growth curve and *Average transpiration rate* (TR) in the well-watered treatment of the experiment in the imaging platform. The graphs show growth curves for lines with the Gladius allele (lower curve) or the Drysdale allele (upper curve) at this locus. Curves are the logistic inference±standard deviation obtained with 1000 bootstrap replicates (function boot in R) on all lines having the considered allele at this locus. Insets show boxplots of TR per unit leaf area for lines with the Gladius (G) or Drysdale (D) allele at this locus. (This figure is available in colour at *JXB* online.)

We found overlaps between QTLs for different variables measured in the platform on three regions of chromosomes 1B, 2A, and 5B (Table 4). On chromosome 1B, the QTL QGRO.atw-1B for Growth_AVE_ and QLER_AVE_.atw-1B.1 for *Average leaf expansion rate* coincided with the QTL peak of QTR.atw-1B for *Average transpiration rate*. These three QTLs carried Drysdale as a positive allele. When looking at pools of RILs carrying Gladius or Drysdale alleles at the marker wsnp_CAP11_c1902_1022590 (Fig. 3), it appeared that the higher transpiration rate and higher growth rate for plants carrying the Drysdale allele were observed in both treatments. This QTL effect seemed intrinsic rather than specific to well-watered conditions.

**Table 4.** Co-localization of QTLs for traits studied in the imaging platform and the polytunnel facility Position on the SSR–DArT–SNP map (1) and SNP map (2). Apd, ACPFG polytunnel drought; apw, ACPFG polytunnel well-watered; 10, year 2010; 11, year 2011; atd, The Plant Accelerator drought; atw, The Plant Accelerator well-watered.. Light-grey shading indicates a positive additive allelic effect (Drysdale); dark grey shading indicates a negative additive allelic effect (Gladius).

Genetic map			Imaging platform QTL						Polytunnel QTL					
Chr	cM (1)	cM (2)	LER_AVE_	Growth._AVE_	LER.Tx	TR	TR	WUE	Yield	Grain number	Grain weight	Harvest index	Spike number	Tiller number
1BL		74.3	QLER_AVE_. atw-1B.2			QTR. atw-1B								
		86.7	QLER_AVE_. atw-1B.1	QGRO. atw-1B									QSnp.apw11-1B	
		112.9												
2AS	88.2								QYie.apw11-2A.2					
	90.9			QGRO. atw-2A.1										
	91.4									QGns.apd10-2A				
2AL	93.4							QWUE.atd-2A	QYie.apw11-2A.1		QGra.apw11-2A			
	109.8			QGRO. atw-2A.2										
4BL	43.3						QTR. atd-4B							
	46.2													
	47.1													QTil.apd10-4B.2
5BL		106.9	QLER_AVE_. atd-5B	QGRO. atd-5B	QTx_LER_. atd-5B							QHar.apw11-5B.1		
		133.1												

### Overlap between QTLs detected with the imaging platform and field trials

In two experiments run in semi-controlled field conditions that included two watering treatments, a total of 84 QTLs (Supplementary Table S1, available at *JXB* online) were found for traits related to biomass, yield and phenology. All QTL identified using the imaging platform, the polytunnel experiments and the field data from Maphosa *et al.* (2014) were positioned on the SSR–DArT–SNP map and the SNP map. This enabled us to identify seven regions with co-located QTLs (Tables 4 and 5).

**Table 5.** Co-localization of QTLs for traits studied in the imaging platform and in the field (Maphosa et al., 2014) Position on the SSR–DArT–SNP map (1) and SNP map (2). Light-grey shading indicates a positive additive allelic effect (Drysdale); dark grey shading indicates a negative additive allelic effect (Gladius).

Genetic map			Imaging platform QTL		Field QTL		
Chr	cM (1)	cM (2)	TR	K_RGR_	Yield	Grain number	Screening
3AL	60.5	116.2					
	62.0						NSW09
	67.2		QTR.atd-3A			NSW10 SAU10 SAU10D	
	67.0	102.4					
	69.4				SAB09 NSW09 SAU10D		
	74.7	94.4					
5AS	21.6				SAR08 SAR09 MEX11	NSW09	
					NSW09	SAU10, SAU10D	
	26.2			QK_RGR_.atr-5A			
	27.6						

A region on chromosome 1B showed QTLs for *Spike number* in the polytunnel (QSnp.apw11-1B) in 2011 and for plant growth-related traits (QGRO.atw-1B and QLER_AVE_.atw-1B.1) and transpiration (QTR.atw-1B) in the platform (Table 4 and Fig. 3). All these QTLs were found in plants grown under well-watered conditions, but some effects could be seen under water deficit. It is worth noting that QGRO.atw-1B, which explained 43% of the genetic variation of *Growth* under well-watered conditions, overlapped with QSnp.apw11-1B, which explained 20% of the variation of *Spike number*.

Two regions of chromosome 2A showed overlapping QTLs between the platform and the polytunnel experiments (Table 4). QGRO.atw-2A.2 for *Average growth rate* in the imaging platform overlapped with three QTLs for *Yield*, *Grain number*, and *Grain weight* in the polytunnel. The second overlap was between two polytunnel QTLs for *Yield* and *Grain weight* and a QTL that explained 9% of the genetic variation of *Average growth rate* in well-watered conditions. It also overlapped with a QTL for WUE in the drought treatment in the platform.

Two QTLs for platform and polytunnel traits were found in close proximity on chromosome 4B (Table 4). The QTL QTR.atd-4B.1 for *Average transpiration rate* was mapped near the QTL for *Tiller number* in the polytunnel, QTil.apd10-4B.2.

Chromosome 5B showed an overlap of loci found in the platform under drought and a QTL controlling *Harvest index* in the polytunnel 2011 experiment (Table 4). Four QTLs associated with *Average leaf expansion rate*, for *T*_*0*_, for leaf expansion curves, and for *Harvest index* were co-mapped on the interval 106.9–133.1 cM of the long arm of this chromosome. Although the *Harvest index* QTL was found for the irrigated treatment, the environmental conditions were hot and dry in 2011 with a maximum VPD of 4.9 kPa in 2011 (vs 3.5 kPa in 2010).

Two regions showed co-location of QTLs from the imaging platform and field when compared with the results of Maphosa *et al.* (2014) (Table 5). A QTL for *Average transpiration rate* in the platform overlapped on chromosome 3A with seven QTLs for yield, grain number, and screenings in the field previously found by Maphosa *et al.* (2014) (Table 5). The transpiration QTL QTR.atd-3A was identified under drought, which matched with the conditions where the yield-related QTLs were expressed: in rain-fed conditions of New South Wales and South Australia, and under cyclic drought in South Australia semi-irrigated conditions.

## Discussion

### Using imaging platforms for analyses of drought responses

One role of imaging platforms, such as The Plant Accelerator, is to enable phenotyping of large numbers of plants using stable environmental conditions and consistent methods across experiments. Previous reports have shown the potential of imaging platforms for QTL mapping and the identification of heritable traits in barley (Chen *et al.*, 2014; Honsdorf *et al.*, 2014). However, for the last 10 years, most studies using such platforms have focused on platform development and analysis procedures (see examples in this special issue), more than application to specific biological questions related to crop improvement. In this study, known procedures of phenotyping have been linked through an operational analysis pipeline to support the genetic analysis of quantitative traits from non-destructive measurements. This allowed not only calculation of dynamic profiles but also derived traits such as soil water potential and transpiration, taking into account the plant weight, and the transpiration per unit leaf area or dynamic WUE.

Although the heritability values were generally low, QTLs of strong effect were found on chromosomes 1A and 1B using the imaging platform that explained between 26 and 43% of the genetic variation (Fig. 3). The G×E interaction could be reduced to some extent by using dynamic variables such as biomass increase and leaf expansion over time in response to the water treatment.

It is worthwhile noting that different sets of QTLs were identified in well-watered and drought conditions. Four QTLs on chromosomes 1A, 2D, and 6B were specifically expressed under well-watered conditions. Five QTLs were found only in the drought treatment of the platform on chromosomes 3A, 4A, 4B, 5B, and 7D and are therefore drought responsive. For fine-mapping and positional cloning, only one watering condition would be required, allowing phenotyping of a large number of lines.

### Genetics of growth, transpiration, and WUE in wheat

To the best of our knowledge, this study is the first to describe QTLs for leaf expansion and plant growth in wheat. Fourteen QTLs were found for growth-related traits. Since several of the variables measured are mathematically dependent, some QTLs are likely to represent the same loci identified using different equations. These QTLs are readily identified since they are located in the same genetic position and show the same additivity. Using these criteria, the total number of loci controlling growth traits in our study is 11.

QTLs for transpiration efficiency have been found previously in wheat (Rebetzke *et al.*, 2008; Wu *et al.*, 2011) using the carbon isotope discrimination method. By comparing our results with those of Rebetzke *et al.* (2008), the QTL QTR.atw-1B of Drysdale/Gladius may be collocated with a QTL for carbon isotope discrimination found on the long arm of chromosome 1B, but more markers will be needed to compare the two map positions. The five QTLs of Drysdale/Gladius for transpiration and WUE on chromosomes 2D, 6A, 3A, 4Bs and 2A were not detected previously by Rebetzke *et al.* (2008) or Wu *et al.* (2011).

Two QTLs for average transpiration rate per unit leaf area (QTR_area_.atw-2D and QTR.atd-4B) were found on chromosomes 2D and 4B. These chromosomes also carry the *Reduced height* (*Rht*) semi-dwarfing genes known to control plant development in wheat (Ellis *et al.*, 2002, 2005). Since TR is calculated from plant biomass, developmental genes, such as the *Rht* genes, are likely to influence TR. However, the locations of *Rht8* (Gasperini *et al.*, 2012) and QTR_area_.atw-2D do not match, and the *Rht1* diagnostic marker (Ellis *et al.*, 2002) was monomorphic in Drysdale/Gladius. Therefore, *Rht* genes do not explain the transpiration QTLs. Further investigations will be needed to identify candidate genes underneath these QTLs.

### Common loci for growth and transpiration rates, and yield components

There were no significant genetic correlations between growth, transpiration, or WUE data from experiments in the platform and yield component traits in the polytunnel. Low correlation is often observed when comparing very different traits (vegetative growth and transpiration vs grain yield) under different growth conditions (glasshouse vs field, and pot vs plot). However, we identified some common QTLs between the platform and the field experiments, which indicate a common genetic basis for both traits. For example, the QTL for biomass increase in pots on chromosomes 1B and 2A under well-watered conditions overlapped with QTLs for yield components in the polytunnel. This indicates that these QTLs might control plant growth across environments, which could be translated into grain yield.

Co-location of QTLs for transpiration traits and yield were also found by Maphosa *et al.* (2014) by comparing their data with the studies of Rebetzke *et al.* (2008) and Wu *et al.* (2011). However, the only co-locations found were in the *Ppd-B1* and *Ppd-D1* regions, which control phenology. This is a well-known drought escape mechanism where the plant cycle is accelerated so that plants flower before the onset of severe drought late in the cropping season. Since these loci are well known, our study aimed to identify loci that were not related to phenology. Consequently, the effects of these two genes were excluded by including their genotypes in the QTL model so that the QTLs we identified are likely to control yield and growth-related traits *per se*.

A locus on chromosome 1B (86.7–112.9 cM) showed three overlapping QTLs controlling LER_AVE_ and Growth_AVE_ in the platform under well-watered conditions, and spike number in the polytunnel in 2011 (Table 4). The higher transpiration rate for plants carrying the Drysdale allele was mainly driven by a higher leaf area and biomass. This QTL seems to be constitutive, as some effects have been found in well-watered as well as under drought conditions (Fig. 3). Several other QTLs for yield and yield components have been detected on chromosome 1B under rain-fed, well-watered, and drought-stressed environments in different wheat mapping populations in Australia, China, India, Mexico, and Spain (Quarrie *et al.*, 2005; Kuchel *et al.*, 2007; Kumar *et al.*, 2007; Maccaferri *et al.*, 2008; Wang *et al.*, 2009; Pinto *et al.*, 2010; Edwards, 2011; Bennett *et al.*, 2012). There is a strong association between the number of stems per plant and relative growth rate in wheat (Dreccer *et al.*, 2013). Our result suggests that the QTL on 1B could control plant growth rate during the vegetative phase, which could affect the number of spikes and grain yield. This QTL would be an interesting target for cloning to identify genes controlling yield across environments in wheat.

## Supplementary data

Supplementary data are available at *JXB* online.

Supplementary Fig. S1. Water-release curve of experiment in the imaging platform.

Supplementary Fig. S2. Correlation between the traits from the platform and the polytunnel experiments.

Supplementary Table S1. QTLs identified for phenology, biomass, and yield-related traits in the polytunnel experiments run in 2010 and 2011 in Urrbrae (South Australia).

Supplementary Data

## Abbreviations:

BIC: Bayesian information criterion
DArT: diversity arrays technology
Growth_AVE_: *Average growth rate*
G×E: genotype by environment
*h*^2^: heritability
K_RER_: *Maximum relative leaf expansion rate*
K_RGR_: *Maximum relative growth rate*
LER: leaf expansion rate
LER_AVE_: *Average leaf expansion rate*
LOD: log of odds
QTL: quantitative trait locus
RER: *Average relative leaf expansion rate*
RGR: *Average relative growth rate*
RIL: recombinant inbred line
SNP: single nucleotide polymorphism
SSR: simple sequence repeat
TR: *Average transpiration rate*
TR_area_: *Average transpiration rate per unit leaf area*
Tx_growth_: *Inflexion point in growth curves*
Tx_LER_: *Inflexion point in leaf expansion curves*
VPD: vapour pressure deficit
WUE: water-use efficiency.
