# Supplementary material for: Combining field performance with controlled environment plant imaging to identify the genetic control of growth and transpiration underlying yield response to water-deficit stress in wheat
Source: J Exp Bot. 2015 Jul 15;66(18):5481–92. doi: 10.1093/jxb/erv320 (PMC4585424; doi:10.1093/jxb/erv320)
Supplement: Supplementary Data [file supp_66_18_5481__index.html]

Combining field performance with controlled environment plant imaging to identify the genetic control of growth and transpiration underlying yield response to water-deficit stress in wheat — Combining field performance with controlled environment plant imaging to identify the genetic control of growth and transpiration underlying yield response to water-deficit stress in wheat — Supplementary Data 

# Combining field performance with controlled environment plant imaging to identify the genetic control of growth and transpiration underlying yield response to water-deficit stress in wheat

## Supplementary Data

Data files

- Supplementary Data - Supplementary Data
- Supplementary Data - Supplementary Data
