# Supplementary material for: Combining field performance with controlled environment plant imaging to identify the genetic control of growth and transpiration underlying yield response to water-deficit stress in wheat
Source: J Exp Bot. 2015 Jul 15;66(18):5481–92. doi: 10.1093/jxb/erv320 (PMC4585424; doi:10.1093/jxb/erv320)
Supplement: Supplementary Data [file supp_erv320_jexbot148254_file002.pdf]

Combining field performance with controlled environment plant imaging to identify the genetic control of growth and transpiration underlying yield response to water deficit stress in wheat

Boris Parent, Fahimeh Shahinnia, Lance Maphosa, Bettina Berger, Huwaida Rabie, Ken Chalmers, Alex Kovalchuk, Peter Langridge, and Delphine Fleury

Supplemental Figures

Supplementary Figure 1

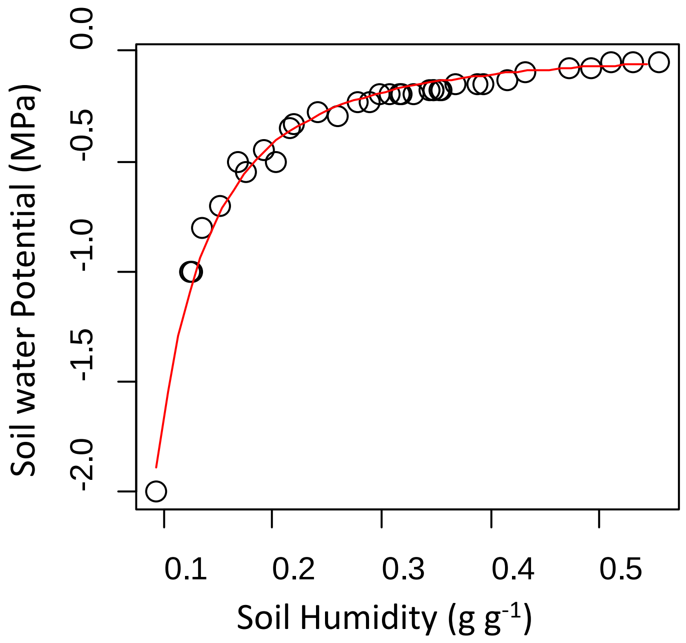

Supplementary Fig.1: *Water release curve of experiment in the imaging platform.*

Soil water content was measured by automatically weighing columns. Predawn leaf water potential was measured on non-expanding leaves, placed into a Scholander-type pressure chamber (Soil Moisture Equipment Corp., Santa Barbara, USA). Soil Humidity was derived from pot weight and soil dry weight. A Van Genucheten curve was fitted to these data.

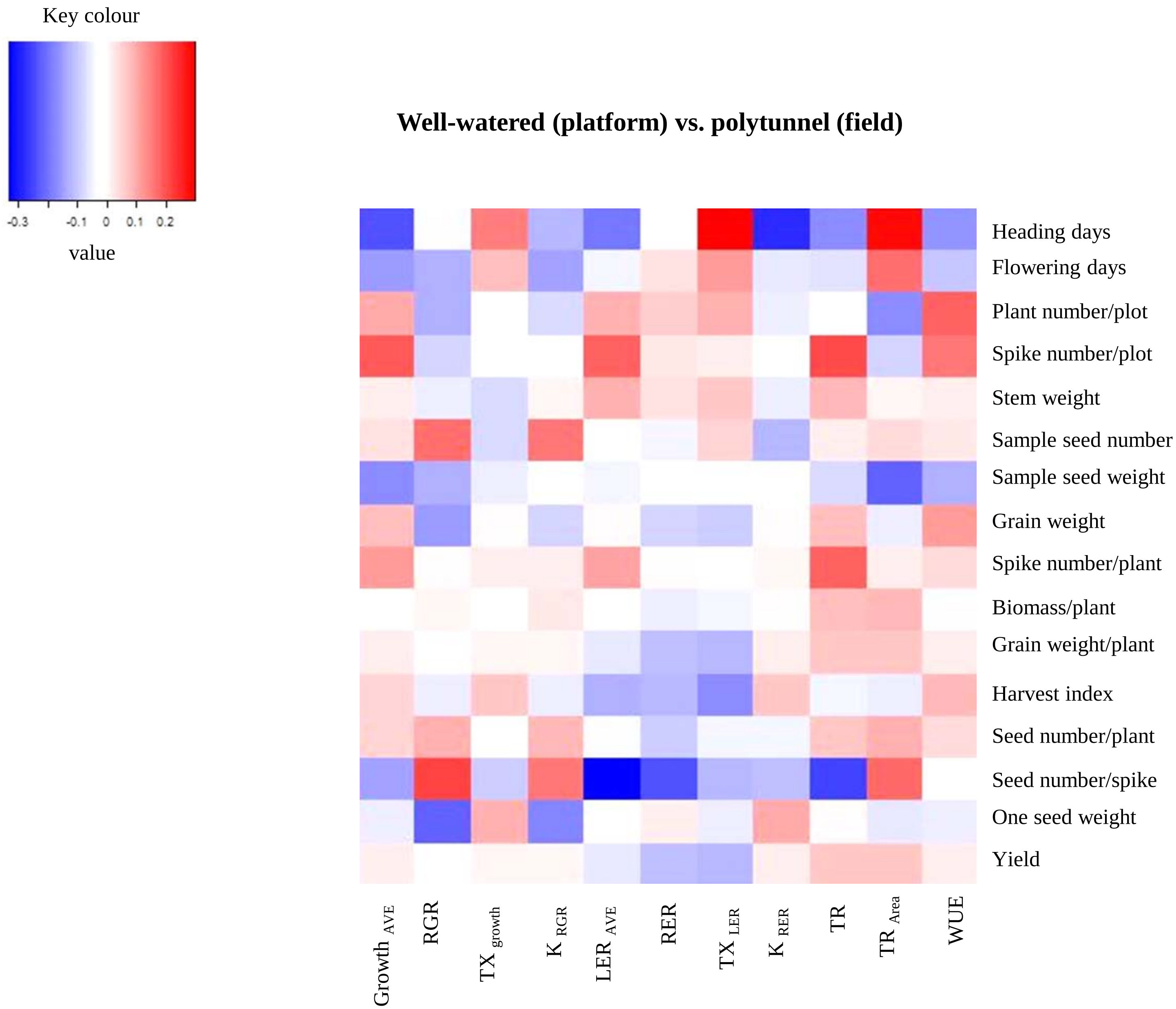
Key colour
-0.3
-0.1
0
0.1
0.2
value
Well-watered (platform) vs. polytunnel (field)
Heading days
Flowering days
Plant number/plot
Spike number/plot
Stem weight
Sample seed number
Sample seed weight
Grain weight
Spike number/plant
Biomass/plant
Grain weight/plant
Harvest index
Seed number/plant
Seed number/spike
One seed weight
Yield
Growth AVE
RGR
TX growth
K RGR
LER AVE
RER
TX LER
K RER
TR
TR Area
WUE

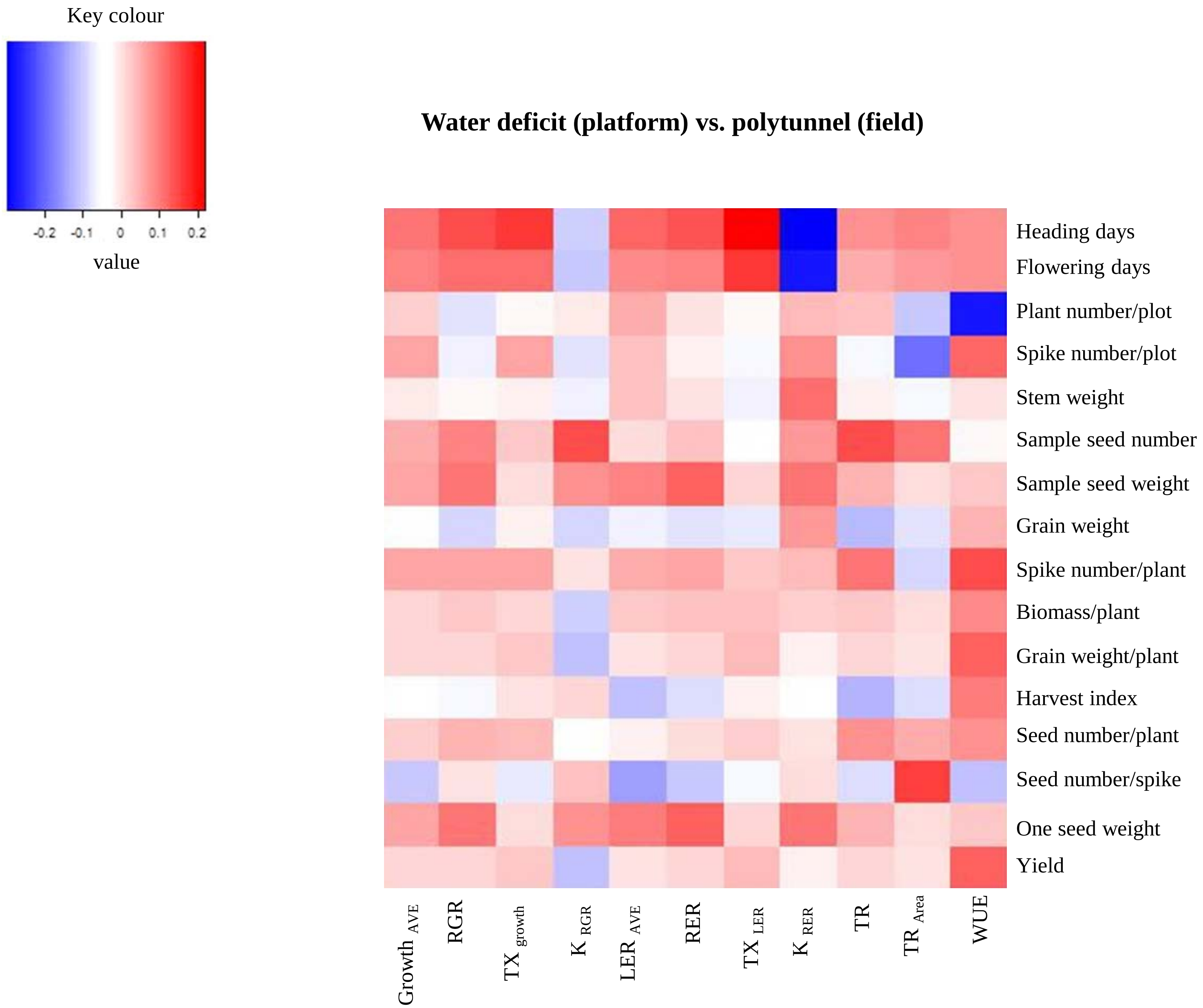
Key colour
-0.2
-0.1
0
0.1
0.2
value
Water deficit (platform) vs. polytunnel (field)
Heading days
Flowering days
Plant number/plot
Spike number/plot
Stem weight
Sample seed number
Sample seed weight
Grain weight
Spike number/plant
Biomass/plant
Grain weight/plant
Harvest index
Seed number/plant
Seed number/spike
One seed weight
Yield
Growth AVE
RGR
TX growth
K RGR
LER AVE
RER
TX LER
K RER
TR
TR Area
WUE
